# Pharmacokinetics Interaction between Imatinib and Genistein in Rats

**DOI:** 10.1155/2015/368976

**Published:** 2015-01-05

**Authors:** Zhe Wang, Li Wang, Meng-ming Xia, Wei Sun, Cheng-ke Huang, Xiao Cui, Guo-xin Hu, Qing-quan Lian, Zeng-shou Wang

**Affiliations:** ^1^Department of Pharmacy, The Second Affiliated Hospital & Yuying Children's Hospital of Wenzhou Medical University, Wenzhou 325027, China; ^2^School of Pharmacy, Wenzhou Medical University, Wenzhou 325035, China; ^3^Zhejiang Province Key Laboratory of Anesthesiology, The Second Affiliated Hospital of Wenzhou Medical University, Wenzhou 325027, China; ^4^Department of Anesthesiology, Critical Care and Pain Medicine, The Second Affiliated Hospital of Wenzhou Medical University, Wenzhou 325027, China

## Abstract

The objective of this work was to investigate the effect of orally administered genistein on the pharmacokinetics of imatinib and N-desmethyl imatinib in rats. Twenty-five healthy male SD (Sprague-Dawley) rats were randomly divided into five groups: A group (control group), B group (multiple dose of 100 mg/kg genistein for consecutive 15 days), C group (multiple dose of 50 mg/kg genistein for consecutive 15 days), D group (a single dose of 100 mg/kg genistein), and E group (a single dose of 50 mg/kg genistein). A single dose of imatinib is administered orally 30 min after administration of genistein (100 mg/kg or 50 mg/kg). The pharmacokinetic parameters of imatinib and N-desmethyl imatinib were calculated by DAS 3.0 software. The multiple dose of 100 mg/kg or 50 mg/kg genistein significantly (*P* < 0.05) decreased the AUC_0−*t*_ and *C*_max_ of imatinib. AUC_0−*t*_ and the *C*_max_ of N-desmethyl imatinib were also increased, but without any significant difference. However, the single dose of 100 mg/kg or 50 mg/kg genistein has no effect on the pharmacokinetics of imatinib and N-desmethyl imatinib. Those results indicated that multiple dose of genistein (100 mg/kg or 50 mg/kg) induces the metabolism of imatinib, while single dose of genistein has no effect.

## 1. Introduction

Imatinib mesylate (Gleevec), a selective tyrosine kinase inhibitor, inhibits BCR-ABL tyrosine kinase activity through selective inhibition of the ATP-binding and then results in inactivation of positive cells [1–3]. In addition, imatinib could competitively inhibit c-KIT receptor tyrosine kinases and platelet-derived growth factor receptor (PDGFR) [1, 4]. Therefore, imatinib was approved for the treatment of chronic myelogenous leukemia (CML) caused by BCR-ABL over expression [5], c-KIT-positive metastatic tumor and gastrointestinal stromal tumor [6]. Moreover, imatinib is approved not only for the treatment of CML and GIST [7], but also for the treatment of dermatofibrosarcoma protuberans (DFSP), myelodysplastic/myeloproliferative diseases (MDS/MPD), aggressive systemic mastocytosis (ASM), hypereosinophilic syndrome/chronic eosinophilic leukemia (HES/CEL) [7–11], and Philadelphia positive acute lymphocytic leukemia (Ph+ALL) [4, 12, 13]. Previous observations indicate that imatinib, a substrate of the cytochrome P 450 isoenzyme cytochrome P3A4 (CYP3A4), is metabolized primarily by CYP3A4 to N-desmethyl imatinib (CGP74588) [3, 14] and to lesser extent by CYP1A2, CYP2C9, CYP2C19, and CYP2D6 [15]. After oral administration, good absorption of imatinib was observed with a bioavailability exceeding 90% [4, 16] and the administered dose (80%) is predominantly metabolized to N-desmethyl imatinib [17]. The pharmacologic activity of N-desmethyl imatinib is similar to that of imatinib, and area under the curve (AUC) plasma concentration against time is approximately 10% to 15% of that for imatinib [17, 18]. Thus, CYP3A4 modulation plays an important role in the pharmacokinetic of imatinib. Recently, interactions based on metabolism have been increasingly reported between drug and natural products. There is great potential for drug-drug or food-drug interactions when imatinib was coadministered with other drugs, foods, and herbal medicine. There was evidence that the AUC of imatinib was observed with approximately 30% reduction when combination with the CYP3A4 inducer St. John's Wort [19]. Conversely, drugs that inhibit CYP3A4 increase imatinib plasma exposure; for example, ketoconazole (CYP3A4 inhibitor) increased the AUC of imatinib approximately 40% with coadministration [4]. Recent studies have shown a correlation between imatinib concentration in plasma and clinical response, suggesting that maintaining imatinib concentrations in plasma at or above the mean concentration of ~1000 ng/mL was important for achieving improved rates of CCR and MMR [20]. Therefore, more attention should be paid to special clinical caution when imatinib is administered with CYP3A4 inhibitor and inducer based on drug interaction.

The use of alternative medicines has been growing increasingly all over the world in the past decade. Soybean was an important part of the diets in many Asian countries. Asian populations are considered to receive significant health benefits from the soybeans in traditional diets due to high contents of isoflavone [21]. Genistein, one of the isoflavonoid compounds, occurs in soybeans and soy-based diets, and this supplements form was nondairy protein component in the diet for their putative health benefits [22–24]. The research indicated that genistein could prevent menopausal symptoms, osteoporosis, cancer, and heart disease [25]. Also, the use of soy products to treat menopause is growing in replacement therapy with the fear of side effects of traditional hormone therapy [24]. Interestingly, the two major isoflavonoids, genistein and daidzein, have been found to exert potential chemoprevention, protection against carcinogenesis and several human cancers [22, 26, 27]. It has been report that CYP1A2, CYP2C8, and CYP3A4 catalyze the reaction in the metabolism of genistein [28, 29]. Furthermore, genistein has inductive or inhibitory effect on the cytochrome P450 (CYP) enzyme system. Evidence has indicated that genistein significantly inhibited CYP1B1 activity and induced CYP3A4 activity at a low or a high level (1 *μ*mol/L or 30 *μ*mol/L) [30]. Therefore, there are potent drug-drug or food-drug interactions when using genistein in combination with other compounds. In addition, the dietary consumption of soy has significant effects on the expression and inducibility of hepatic CYP450 enzymes in rat, especially the CYP3A [31]. However, it is not presently clear whether genistein has the capacity to affect the pharmacokinetics of imatinib.

In this study, the pharmacokinetics of imatinib and N-desmethyl imatinib in rats were investigated after administration of genistein by simultaneous determination of imatinib and N-desmethyl imatinib in rat plasma with an ultrahigh performance liquid chromatography-mass spectrometry method.

## 2. Material and Methods

### 2.1. Chemicals and Reagents

Imatinib mesylate (purity > 99%) was purchased from SellecK Chemicals LLC (Houston, USA). N-Desmethyl imatinib (purity > 98%) was purchased from Toronto Research Chemicals Inc. (North York, Canada). Carbamazepine (purity > 98.0%) was purchased from the National Institute for the Control of Pharmaceutical and Biological Products (Beijing, China) and used as the internal standard (IS). Genistein (purity > 99%) was purchased from commercial sources (INDOFINE Chemical Company, Inc., Somerville, NJ, USA). Acetonitrile and methanol were acquired from the Merck Co. (Darmstadt, Germany). All other chemicals for this study were reagent grade and used without further purification.

### 2.2. Equipment and UPLC-MS/MS Analysis

Imatinib and N-desmethyl imatinib plasma concentrations were determined by ultrahigh performance liquid chromatography-mass spectrometry method (UPLC-MS/MS). UPLC–MS/MS analyses were performed by an Acquity UPLC Xevo TQ triple quadrupole mass spectrometer (Waters Co., Milford, MA) equipped with an electrospray ion source. Chromatographic separation was performed using a Waters ACQUITY BEH C18 column (50 mm × 2.1 mm i.d., 1.7 *μ*m particle size) thermostated at 25°C. The mobile phase was composed of 0.1% formic acid (A) and acetonitrile (B) with gradient as follows: 0.0–0.5 min at 16.5% B, 0.5–2.00 min linear increase to 98% B, 2.00–2.50 min at 98% B, and 2.50–3.50 min at 16.5% B. And the flow rate was 0.4 mL/min. The total run time was 3.5 min. The electrospray interface was maintained at 500°C. Nitrogen nebulization was performed with a nitrogen flow of 800 L/h. Argon was used as the collision gas. Imatinib, N-desmethyl imatinib, and IS were detected in multiple reaction monitoring (MRM) scan mode with positive ion detection. The precursor-product ion pairs used for the MRM detection were m/z 494.2 → 394.2 for imatinib, m/z 480.2 → 394.2 for N-desmethyl imatinib, and m/z 237.1 → 194.2 for carbamazepine (IS).

### 2.3. Sample Preparation

In a 1.5 mL centrifuge tube, an aliquot of 10 *μ*L of IS working solution (1 *μ*g·mL^−1^) was added to 0.1 mL of collected plasma sample and followed by the addition of 0.3 mL of acetonitrile. The tubes were vortex mixed for 0.5 min. After centrifugation at 15,000 rpm for 10 min, 50 *μ*L of supernatant was transferred into a clean tube and mixed with 50 *μ*L of ultrapure water. Next, 6 *μ*L of the mixture was injected into the LC-MS/MS system for analysis.

### 2.4. Animals and Treatment

Male Sprague-Dawley rats with body weights of 250 ± 20 g were purchased from Shanghai SLAC Laboratory Animal Co., Ltd. (Certificate no. 2007-0005). The rats were acclimatized for a week in laboratory conditions to minimize all efforts of any animal suffering before initiating the experiment. Necessary approval from the Institutional Animal Ethics Committee was obtained to carry out the experiments.

### 2.5. Pharmacokinetic Experiment

Twenty-five male Sprague-Dawley rats were randomly divided into five groups (five rats for each group): A group (the control group), B group (the multiple dose of 100 mg/kg genistein for consecutive 15 days), C group (the multiple dose of 50 mg/kg genistein for consecutive 15 days), D group (a single dose of 100 mg/kg genistein), and E group (a single dose of 50 mg/kg genistein). After the last administration of genistein or 0.5% CMC-Na (control group), a single dose of 30 mg/kg imatinib was administered orally to all rats in each group. Blood samples (300 *μ*L) were directly collected into a clean tube through the tail vein at 0 (predose), 0.5, 1, 2, 3, 4, 6, 8, 12, and 24 h after imatinib oral administration. And blood samples were immediately separated by centrifuging at 5000 rpm for 10 min, and 100 *μ*L plasma samples were transferred to another tube and kept frozen at −80°C until being analyzed.

### 2.6. Statistical Analysis

The results are given as mean standard deviation (SD). The noncompartmental analysis was used to calculate the pharmacokinetic parameters by DAS version 3.0 (Bontz Inc., Beijing, China). The statistical analyses were evaluated by one-way ANOVA and a Dunnett's multiple range test for the unpaired data (SPSS 19.0, Chicago, IL). A value of *P* < 0.05 was considered to be statistically significant.

## 3. Results

### 3.1. UPLC-MS/MS Analyses

**Figure 1:**
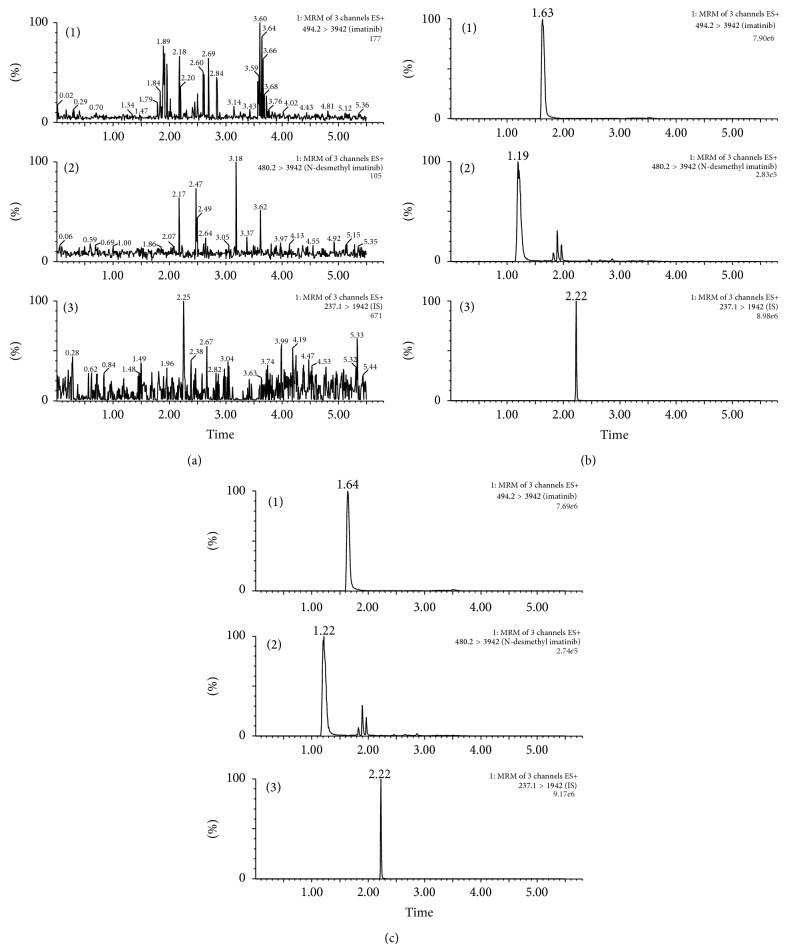
Extracted ion chromatograms for (a) blank rat plasma, (b) blank rat plasma spiked with imatinib (10 μg/mL), N-desmethyl imatinib (0.500 μg/mL), and IS (carbamazepine), and (c) rat plasma sample after oral administration of single dosage 30 mg/kg imatinib. (1) Imatinib, (2) N-desmethyl imatinib, and (3) IS (carbamazepine).

No interference can be observed in the UPLC chromatograms. The retention times of imatinib, N-desmethyl imatinib, and internal standard were approximately 1.19, 1.63, and 2.22 min, respectively.  Figure 1 shows the typical chromatograms of a blank plasma sample, a blank plasma sample spiked with imatinib, N-desmethyl imatinib, and IS, and a plasma sample after oral administration of imatinib. The calibration curves of imatinib and N-desmethyl imatinib were linear at a concentration range of 0.10–20 *μ*g·mL^−1^ for imatinib and 0.05–1.00 *μ*g·mL^−1^ for N-desmethyl imatinib, both with a correlation coefficient *r* = 0.997. RSD of intraday and interday precision was in the range of 3.12%–6.42% for imatinib and 2.21%–11.40% for N-desmethyl imatinib, respectively. And the accuracy of the method ranged from 98.85% to 107.34% for imatinib and 94.60% to 105.95% for N-desmethyl imatinib, respectively. The above results demonstrated that the values were within the acceptable range and the method was accurate and precise.

### 3.2. Effects of Multiple Dose of 100 or 50 mg/kg Genistein on Imatinib and N-Desmethyl Imatinib

**Figure 2:**
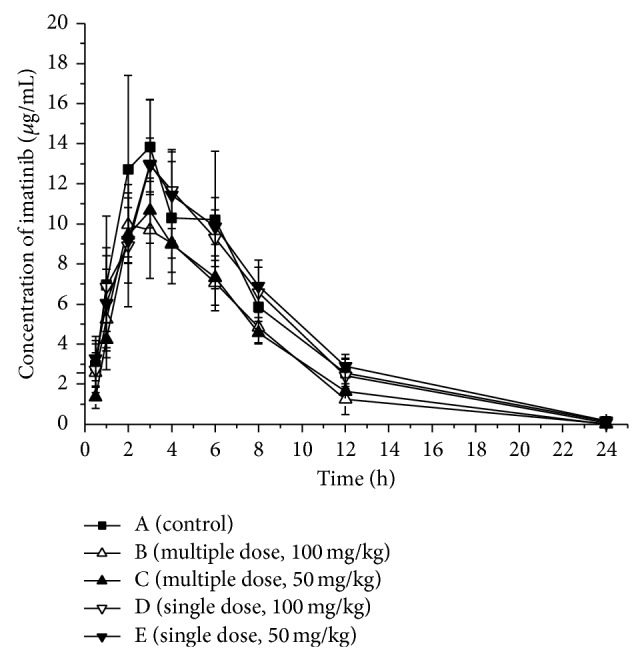
Mean concentration-time curve of imatinib in five groups (*n* = 5).

**Figure 3:**
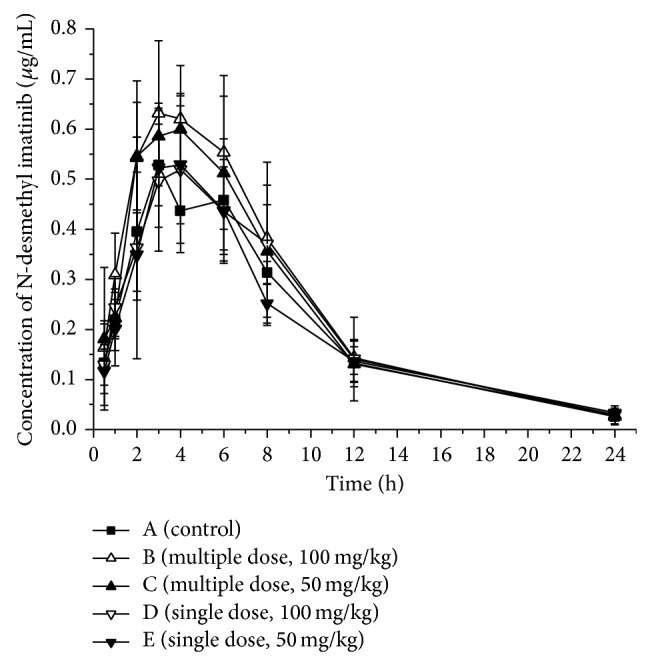
Mean concentration-time curve of N-desmethyl imatinib in five groups (*n* = 5).

The mean pharmacokinetic parameters of imatinib and N-desmethyl imatinib administered alone or in combination with 100 mg/kg (Group B) and 50 mg/kg (Group C) genistein and the statistical test results are presented in Tables 1 and 2. Mean plasma concentration-time curves of imatinib and N-desmethyl imatinib in different groups are presented in Figures 2 and 3.

After pretreatment with a multiple dose of genistein, mean peak plasma concentrations (*C*_max⁡_) and AUC of imatinib were significantly decreased compared to those of the control group (11.036 *μ*g·mL^−1^, 10.810 *μ*g·mL^−1^ versus 14.511 *μ*g·mL^−1^; group B, group C versus control group). However, the time to reach the peak plasma concentration (*T*_max⁡_), the half-life, and mean residence time (MRT) were not significantly different between the treatment groups. On the contrary, the main pharmacokinetic parameters of N-desmethyl imatinib had no great difference between the control and treatment groups. The results indicated that genistein had the potential to increase rat metabolism rate of imatinib* in vivo* after the multiple dose treatment.

### 3.3. Effects of a Single Dose of 50 or 100 mg/kg Genistein on Imatinib and N-Desmethyl Imatinib

The mean pharmacokinetic parameters of imatinib and N-desmethyl imatinib administered alone or in combination with 100 mg/kg (Group D) and 50 mg/kg (Group E) genistein and the statistical test results are presented in Tables 1 and 2. Mean plasma concentration-time curves of imatinib and N-desmethyl imatinib in different groups are presented in Figures 2 and 3.

Compared with the control group, the main pharmacokinetic parameters of imatinib after a single dose of genistein were not significantly decreased or increased. Similarly, the main pharmacokinetic parameters of N-desmethyl imatinib had no great difference between the control and the treatment groups.

## 4. Discussion

Imatinib is metabolized primarily to N-desmethyl imatinib by CYP3A4, which is known as the rate-limiting step in the metabolism [32]. Drugs that inhibit or induce CYP3A4 activity in metabolizing could alter imatinib pharmacokinetics. Natural products, such as flavone, isoflavones, and tangeretin, could affect the human CYP activities when administered orally [33]. A previous study in our laboratory indicated that the coadministration with apigenin, one of the flavonoid compounds, would improve the concentrations of imatinib in plasma with multiple dose of apigenin administrations but reduce the concentrations with single dose administration [34]. In this study, the results indicated that multiple doses of genistein administrations in rat could influence the pharmacokinetics of imatinib. There was no systemic metabolic drug-drug interaction, as the *t*_1/2_ and MRT of imatinib were not significantly changed. However, imatinib exposure (*C*_max⁡_, AUC) was reduced by multiple doses of genistein administration. It indicates that there was a presystemic metabolic drug-drug interaction [35]. The influence of 50 mg/kg multiple dose of genistein group was similar with that of 100 mg/kg multiple dose group. There was no significant difference found between the two treatment groups (B and C), suggesting that the inductive effect was not dose dependent. However, the absence of difference could be due to the low number of rats in each group. The AUC of main metabolite N-desmethyl imatinib was increased as well as *C*_max⁡_. This further explains inductive effect on CYP3A4 by genistein.

There is a correlation between imatinib trough plasma level and clinical response. Therefore, low imatinib plasma levels may result in treatment failure [20]. The inductive effect on CYP3A4-mediated metabolism of low genistein concentrations has been previously demonstrated* in vitro* [30], and it was most likely due to increasing expression of hepatic CYP3A [36]. It is well known that CYP3A gene is regulated by pregnane X receptor (PXR), which plays an important role in the regulation of xenobiotic-inducible CYP3A gene expression, and genistein can significantly transactivate full-length, wild-type human PXR and induce the activity of the human CYP3A4 luciferase reporter [22]. In this study, we could infer that the CYP3A4 enzyme was induced under the influence of genistein after multiple dose of imatinib, as the AUC and *C*_max⁡_ of imatinib were decreased; meanwhile, exposure of N-desmethyl imatinib was decreased.

In addition, genistein did not exert any inductive or inhibitory effect on the pharmacokinetics of imatinib at a single dose. This result may be due to the poor absorption and rapid metabolism of genistein after a single dose.* In vitro* studies demonstrated that genistein does not inhibit CYP3A5-mediated metabolism of the marker substrate [37]. Furthermore, our previous study suggested that genistein modestly inhibits CYP3A activity and only at a high level could reduce the conversion of imatinib to N-desmethyl imatinib in rat liver microsomes. Thus, no inhibitory effect on imatinib was observed due to its weakly inhibitory effect of genistein on CYP3A4.

## 5. Conclusion

The intake of genistein may induce the activity of CYP3A4 and consequently decrease imatinib plasma levels and some of its pharmacokinetic parameters (AUC_0–*t*_, *C*_max⁡_). However, the clinical significance of the pharmacokinetic interaction between imatinib and genistein has to be confirmed through further studies.

## Figures and Tables

**Table 1:** The main pharmacokinetic parameters of imatinib in five groups (*n* = 5).

Parameters	A	B	C	D	E
*C*_max⁡_ (mg·L^−1^)	14.511 ± 2.821	11.036 ± 1.681^*^	10.810 ± 1.685^*^	12.936 ± 1.348	13.136 ± 3.116
*t*_1/2_ (h)	2.899 ± 0.864	2.298 ± 0.608	2.200 ± 0.604	2.173 ± 1.010	3.315 ± 0.288
*T*_max⁡_ (h)	2.600 ± 0.548	2.400 ± 0.548	2.800 ± 0.447	3.000	3.400 ± 0.548
Vz/*F* (L·kg^−1^)	1227.409 ± 323.953	1347.24 ± 263.74	1288.052 ± 369.454	978.843 ± 513.082	1418.396 ± 348.368
CLz/*F* (L·kg^−1^)	300.125 ± 49.474	413.894 ± 41.234	406.776 ± 30.891	311.401 ± 43.075	295.466 ± 59.811
AUC(0–*t*) (*μ*g·h·L^−1^)	108.145 ± 18.062	77.741 ± 7.592^*^	78.914 ± 6.625^*^	104.072 ± 15.944	110.164 ± 18.592
AUC(0–*∞*) (mg·h·L^−1^)	109.010 ± 18.343	77.932 ± 7.766^*^	79.070 ± 6.635^*^	104.491 ± 15.695	1111.310 ± 18.701
MRT(0–*t*) (h)	6.182 ± 0.509	5.561 ± 0.736	5.877 ± 0.140	6.206 ± 0.724	6.538 ± 0.334
MRT(0–*∞*) (h)	6.356 ± 0.693	5.611 ± 0.770	5.922 ± 0.100	6.301 ± 0.874	6.781 ± 0.409

^*^ *P* < 0.05 = significance in comparison to group A (ANOVA).

**Table 2:** The main pharmacokinetic parameters of N-desmethyl imatinib in five groups (*n* = 5).

Parameters	A	B	C	D	E
*C*_max⁡_ (mg·L^−1^)	0.566 ± 0.063	0.694 ± 0.111	0.658 ± 0.044	0.546 ± 0.145	0.552 ± 0.110
*t*_1/2_ (h)	4.099 ± 1.415	3.864 ± 1.563	3.857 ± 0.949	4.559 ± 0.605	4.736 ± 1.323
*T*_max⁡_ (h)	3.800 ± 1.304	4.400 ± 1.517	4.000 ± 1.871	4.600 ± 1.949	4.600 ± 1.342
Vz/*F* (L·kg^−1^)	37429.442 ± 12484.149	29407.106 ± 12819.755	30990.850 ± 5752.33	42622.588 ± 14954.107	44070.769 ± 13901.919
CLz/*F* (L·kg^−1^)	6435.171 ± 797.154	5250.116 ± 512.648	5709.087 ± 941.271	6374.325 ± 1785.552	6459.427 ± 760.373
AUC(0–*t*) (mg·h·L^−1^)	4.890 ± 0.573	5.978 ± 0.680	5.608 ± 0.951	5.178 ± 1.165	4.798 ± 0.575
AUC(0–*∞*) (mg·h·L^−1^)	5.037 ± 0.650	6.144 ± 0.629	5.748 ± 1.087	5.380 ± 1.617	5.007 ± 0.571
MRT(0–*t*) (h)	7.088 ± 0.548	6.751 ± 0.659	6.762 ± 0.518	7.205 ± 0.757	7.276 ± 0.668
MRT(0–*∞*) (h)	7.752 ± 1.062	7.415 ± 1.180	7.271 ± 0.925	8.152 ± 0.800	8.300 ± 1.219
